# Barriers and facilitators to paediatric caregivers’ participation in virtual speech, language, and hearing services: A scoping review

**DOI:** 10.1177/20552076231216684

**Published:** 2023-11-29

**Authors:** Danielle DiFabio, Sheila Moodie, Robin O’Hagan, Simrin Pardal, Danielle Glista

**Affiliations:** 1 School of Health and Rehabilitation Sciences, 6221 Western University, London, ON, Canada; 2 The National Centre for Audiology, 6221 Western University, London, ON, Canada; 3 The School of Communication Sciences and Disorders, 6221 Western University, London, ON, Canada; 4 School of Health Studies, 6221 Western University, London, ON, Canada

**Keywords:** Caregivers, virtual care, participation, family-centered care, audiology, speech-language pathology

## Abstract

**Purpose:**

Virtual care-related technologies are transforming the way in which health services are delivered. A growing number of studies support the use of virtual care in the field of audiology and speech-language pathology; however, there remains a need to identify and understand what influences caregiver participation within the care that is virtual and family-focused. This review aimed to identify, synthesize, and summarize the literature around the reported barriers and facilitators to caregiver participation in virtual speech/hearing assessment and/or intervention appointments for their child.

**Methods:**

A scoping review was conducted following the Joanna Briggs Institute manual for evidence synthesis. A search was conducted using six databases including MEDLINE, CINAHL, SCOPUS, ERIC, Nursing and Allied Health, and Web of Science to collect peer-reviewed studies of interest. Data was extracted according to a protocol published on Figshare, outlining a predefined data extraction form and search strategy.

**Results:**

A variety of service delivery models and technology requirements were identified across the 48 included studies. Caregiver participation was found to vary across levels of attendance and involvement according to eight categories: Attitudes, child behavioral considerations, environment, opportunities, provider-family relationship, role in care process, support, and technology.

**Conclusions:**

This review presents a description of the key categories reported to influence caregiver participation in virtual care appointments. Future research is needed to explore how the findings can be used within family-centered care models to provide strategic support benefiting the use and outcomes of virtual care.

## Introduction

Virtual care related technologies are transforming the way in which health services are delivered. *Virtual care* is defined as any interaction between a provider and their patients and/or members of their circle of care, occurring remotely, and using any form of communication or information technology with the aim of facilitating or maximizing the quality and effectiveness of patient care.^1,2^ This broad definition can be applied to many types of health care interactions and involve various members of the care process. Virtual care can be tailored according to care needs and preferences and can facilitate synchronous (real-time interaction including face-to-face videoconferencing), asynchronous (collecting, storing, and forwarding information, including the use of email), or hybrid interactions (including any combination of synchronous, asynchronous, and/or in-person delivery).^1–3^ For virtual care to be successfully implemented in clinical practice there are a variety considerations, such as the barriers and facilitators related to technology and infrastructure requirements, and the availability of best-practice guidelines for clinicians related to virtual care delivery.^4–6^

Healthcare providers working in the field of communication sciences and disorders deliver care related to impairments in hearing, language, and/or speech processes. This care includes the delivery of early hearing detection and intervention programmes to ensure timely diagnosis of permanent hearing loss to support foundational early language development.^7–10^ Early intervention in a child's language development is important for optimal outcomes in communication, cognition, social communication, social-emotional functioning, pragmatics, and academic success.^11–16^ Best practices in family-centered early intervention for families with children who receive audiology and/or speech-language pathology (SLP) services highlight the need for collaborative, supportive, and engaged family participation for optimal child development outcomes.^14,15,17–22^ There is growing evidence demonstrating virtual care as an accepted method of care provision for paediatric, speech, language, and hearing services.^23–28^ Despite the growing evidence, the development of evidence-informed guidance around the provision of virtual care targeting specific patient populations, such as paediatrics, is needed to support sustainable best-practice implementation.

In paediatric interventions, caregiver participation includes various levels of *attendance* and/or *involvement* to facilitate supportive, encouraging, and family-centered care delivery. *Attendance,* a prerequisite for involvement, relates to the individual's physical presence during the intervention, while *involvement* moves beyond physical attendance and includes the experience of actively engaging in the intervention.^29,30^ Participation can be viewed as both a process and an outcome. When considered a process, participation is thought of as an entry-point that guides the intervention and/or outcome.^30^ *Involvement*, commonly referred to as engagement, describes the experience of participation; it is a construct expressed at multiple levels of human functioning.^30^

There are a multitude of components cited in the literature that have been found to influence the overall success of virtual care in audiology and SLP.^31–34^ These components include technological influences, such as access to technology or technology difficulties, and the digital divide between those who have access to technologies and those who do not, which most negatively impacts ethnic minorities, individuals of low socioeconomic status, and those in rural areas. Additionally, the cost associated with care provision (e.g., upfront costs associated with providing virtual care for providers, costs incurred by patients to access technology and the reduction of travel-related costs for families), travel (e.g., elimination of travel time for families, and the reduction of challenges relating to travel for patients with mobility challenges), and beliefs about the technology capabilities of families. Overall, these considerations can differ depending on geographical location along with internet access and resource availability, as key considerations.

Despite the growing number of professional practice guidelines and protocols for virtual audiology and SLP care, existing literature has yet to comprehensively identify what influences paediatric caregiver participation (attendance and involvement) in virtual speech, language, and hearing care.^3,35,36^ Scoping review methodology can be used to investigate and chart current knowledge about a topic and to identify existing knowledge gaps for future investigation.^37,38^ This scoping review included the following objectives:

1. Identify, synthesize, and summarize peer-reviewed literature surrounding the barriers and facilitators influencing caregiver participation in virtual care related to their child(ren)'s audiology and/or SLP assessment or intervention appointments.
2. Describe the reported levels of caregiver participation related to type of caregiver activities across audiology and/or SLP interventions.
3. Categorize the identified barriers and/or facilitators to caregiver participation.

Findings from this review may help inform future implementation of virtual care for paediatric speech, language, and hearing services that incorporate caregivers into the care process and identify strategies to improve participation in appointments.

## Methods

This review followed the recommendations of the Joanna Briggs Institute (JBI) methodology for scoping reviews and is reported using the Preferred Reporting Items for Systematic reviews and Meta-Analyses extension for Scoping Reviews (PRISMA-ScR) guidance and checklist.^39,40^

### Protocol and registration

Prior to initiating the scoping review process, the research team developed a protocol document. This has been made publicly available on Figshare.com and includes the full search strategy across all included databases.^41^ The PRISMA-ScR checklist is available as a Supplemental Material (Supplement A). This review did not require ethics approval as scoping review projects are exempt from the research ethics review process based on the use of secondary and anonymized information.^42^

### Eligibility criteria

Studies were included using the following criteria: (a) peer-reviewed sources; (b) published or accessible in English; (c) care was delivered virtually (or used a phone call as a backup communication method, in the case of technical difficulties); (d) studies related to speech, language, and/or hearing intervention and/or assessments; (e) specific to care provided to children; (f) care delivery led by a licensed healthcare professional; and (g) with a participating caregiver. Studies that used student clinicians to deliver the virtual care appointment were included only if they were supervised and/or guided by a licensed health care provider during virtual care. Studies not published or available in English were excluded based on the research team's language limitations. Grey literature was excluded for the purpose of this review. Studies that used a telephone audio-only call as the only delivery model were excluded to align with optimal communication methods for speech, language, and hearing care, specific to face-to-face virtual communication achieved through the use of video modalities to provide visual and non-verbal cues.^3,43,44^

### Information sources

This study utilized six databases, MEDLINE (OVID), CINAHL (EBSCO), SCOPUS (Elsevier), ERIC (IES), Nursing and Allied Health (EBSCO), and Web of Science (Clarivate). When selecting databases, scoping review protocols recommend a minimum of two relevant databases, highlighting the importance of using a combination of databases, with specific attention to use MEDLINE, CINAHL, and Web of Science databases.^39,45^ Databases were selected following the recommendations of a research librarian at Western University, with the aim of being as comprehensive as possible. There were no limits placed on the study context or date range of studies included. The database search was initiated in June 2021 and repeated in October 2021 to identify additional articles. During the full-text screening phase, one reviewer (DD) underwent additional attempts to access articles that were identified as not having full-text availability using Western University's library database and by contacting the respective first authors through Research Gate or via email, when accessible.

### Search terms

Three authors (DD, DG, SM) constructed a list of preliminary search terms. These were revised with support from a research and scholarly communications librarian to include text words specific to subject headings, keywords (e.g., virtual, health care, caregiver, child, audiology, and SLP), and indexing terms (e.g., Medical Subject Headings). Appendix A provides a list of all the search terms used in the database search strategy for Ovid MEDLINE. The search strategy was replicated and modified for the remaining databases. Appendix A provides a list of all the search terms used in the database search strategy for Ovid MEDLINE. These terms were used as part of the search strategy, replicated for the remaining databases, and modified in the case of the use of alternate Boolean operators.

### Selection of sources of evidence

A three-step search strategy was used in the scoping review protocol.^39,46^ Step 1 (initial search), Step 2 (modification of search strategy following by search and screen processes), and Step 3 (identifying any additional sources of evidence).

*Step 1*. An initial limited search, with at least two appropriate online databases (Ovid MEDLINE and CINAHL [EBSCO] databases). This initial limited search was conducted by one member of the research team (DD).
*Step 2*. An analysis of text words contained in the title, abstract of the initial search, and of the index terms used to describe the articles; followed by a final search using all identified key words and indexed terms, across all included databases. All records were imported and organized using Covidence online software, for systematic review management.^47^ During import of database searches into Covidence, Covidence automatically identified and removed duplicate articles. A pilot screening of 25 randomly selected articles was conducted by two team members (DD, SP) to ensure eligibility criteria were consistently applied. During the screening phases, disagreements over eligibility of a study's inclusion in the review were addressed by a senior team member (SM) during team meetings. Two members of the research team (DD, SP) independently completed title and abstract screenings using Covidence. When differences of opinions arose for the inclusion of specific studies, team meetings (DD, SP, SM) were held to reach consensus. Full-text screening procedures began with a second calibration exercise for the two reviewers (DD, SP), where they screened five randomly selected full-text studies and discussed any discrepancies following the same process used during title and abstract screening.
*Step 3*. Examination of the reference lists of all identified sources. During the data extraction phase hand-picking of studies from scholarly publication updates and referenced citations was conducted.

### Data charting process and data items

The research team developed a draft data charting table to guide data extraction (Supplement B), using Microsoft Excel (Version 16.64) for team contribution to data extraction. The data extraction phase included all members of the research team (DD, DG, RO, SM, SP). To standardize data extraction and reach consensus on extraction components, the extraction form was piloted by all authors using two randomly selected studies. The final data charting table included predefined components of interest specific to study design, participant details, type of intervention, measures, and outcomes. Two members of the research team were assigned to each article, with the lead reviewer (DD) assigned all articles and a second reviewer (DG, RO, SM, SP) assigned to approximately one-quarter of the included articles. Each team member independently conducted data extraction using Excel. Differences in opinions were discussed during regularly scheduled team meetings. Data extraction was iterative, with categories added or amended in accordance with the aims of the review and with agreement from all authors.

### Synthesis of results

Upon completion of final data extraction, data underwent inductive content analysis to identify barriers and/or facilitators to paediatric caregiver's participation in virtual speech, language, and hearing services. Inductive content analysis is a method for making replicable and valid interpretations from data to their context, where categories are derived from the data to provide new knowledge, insights, and a guide to action.^48,49^ During the data extraction process, the team identified barriers and facilitators to caregiver participation which were analyzed into various categories, outlined in the results section. Relationships between categories were identified and used to group categories and their related sub-categories, thus moving the data from specific to general (e.g., the main category *environment* included the sub-categories *contextual environment* and *family involvement*).^50^

## Results

**Figure 1.**
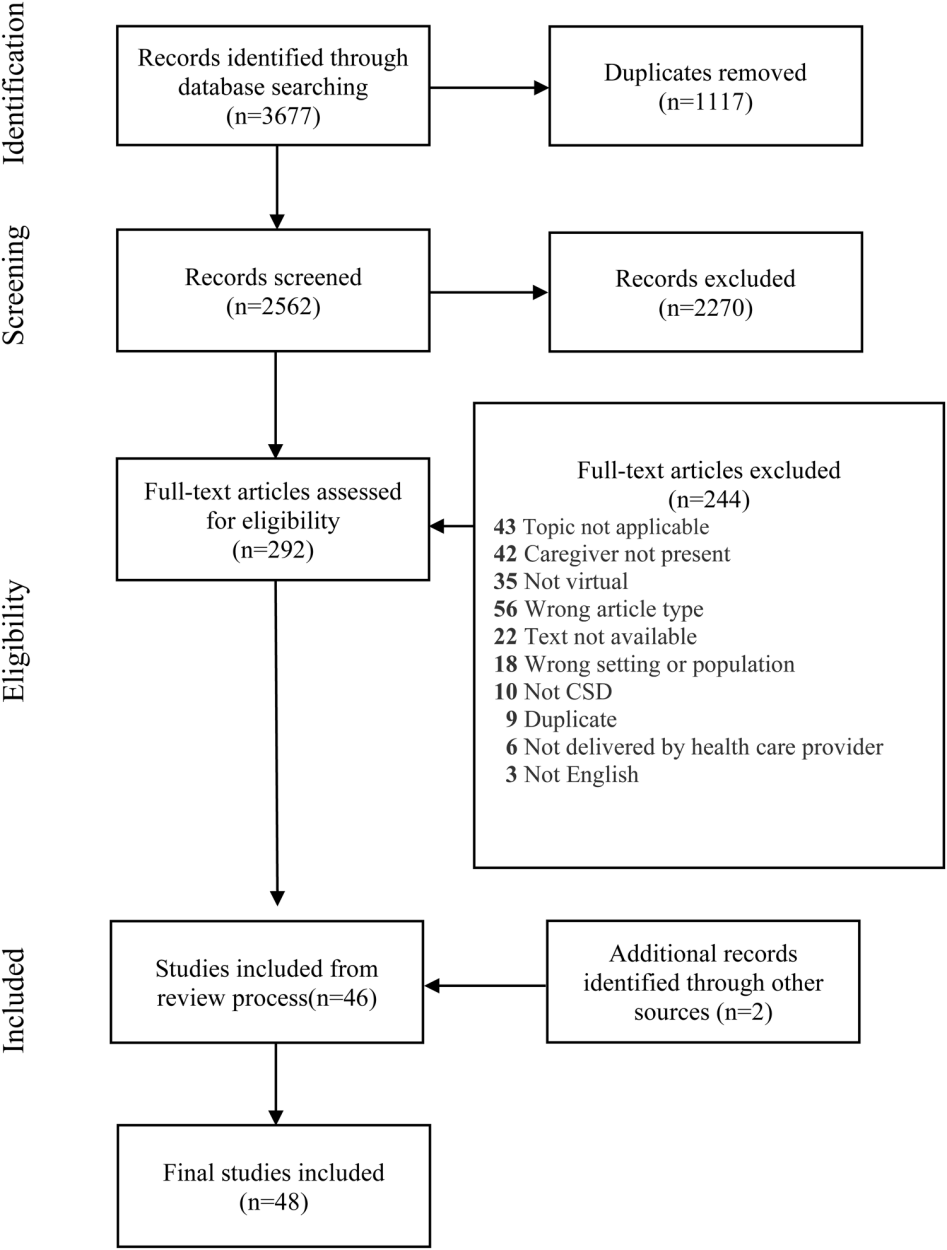
Preferred Reporting Items for Systematic reviews and Meta-Analyses extension for Scoping Reviews (PRISMA-ScR) flow diagram.

A total of 3677 studies were identified through the database searches, with 1117 duplicates removed. During the title and abstract screening phase, two reviewers (DD, SP) removed 2270 studies based on the eligibility criteria. A total of 244 studies were removed during the full-text review phase in alignment with the following criteria: Topic not applicable, caregiver not present, care delivery not virtual, wrong article type (i.e., not containing an intervention or assessment), text not available, wrong setting or population, not audiology or SLP, duplicate article, care not delivered by a healthcare provider, and/or not written or accessible in English. The process for exclusion and inclusion of the studies for this review have been summarized in a PRISMA-ScR flow diagram, Figure 1.

### Overview of the included studies

A total of 46 studies were identified during the screening phases, and an additional two articles were identified though handpicking and review of reference lists of included articles. Of the articles included in this review, 36 studies were published prior to the onset of the COVID-19 pandemic (i.e., earlier than 2020). The 12 studies published during the pandemic (between 2020 and 2021), included three studies that reported data collection occurring during the pandemic, three reported data collection occurring prior to the pandemic, and the remaining six studies did not include details surrounding data collection timelines. Studies used a variety of data collection methods, including interview (*n* = 17) or survey-based (*n* = 32) methods, video analysis (*n* = 6), observation (*n* = 5), audio analysis (*n* = 2), and/or case reports (*n* = 2). Over 80% of the studies from this review were conducted in the Global North, which includes the United States (*n* = 23), Canada (*n* = 2), United Kingdom (*n* = 3), Australia (*n* = 14), and South Korea (*n* = 1).^51^ The remaining studies were conducted in the Global South, including Mexico (*n* = 2), South Africa (*n* = 1), Taiwan (*n* = 1), and South India (*n* = 1).^51^

Thirty-four studies facilitated virtual follow-up appointments, eight studies used virtual care as their initial appointment, three studies included both initial and follow-up virtual appointments, and three studies did not clearly state if the appointments were initial or follow-up. Many included studies were related to SLP interventions (*n* = 29), with the remaining studies either related to audiology (*n* = 15) or both SLP and audiology (*n* = 4). In all studies, a healthcare professional was the primary care provider, with the majority led by speech-language pathologists (*n* = 26), followed by audiologists (*n* = 9), or other healthcare professionals (*n* = 13).

The delivery models used across the included studies were varied. Twenty-three studies used synchronous videoconferencing, two studies used asynchronous models (including applications, asynchronous videos, or phone), and the remaining studies used hybrid models of care. A variety of hybrid models were reported. The most frequent hybrid model included synchronous videoconferencing paired with in-person care (*n* = 11). Additional hybrid models encompassed synchronous with asynchronous components, asynchronous with in-person components, or a combination of all three types of care (synchronous, asynchronous, and in-person). One study allowed the caregiver to select their preferred care delivery method.^52^ In addition to care delivery model variations, the physical location of the caregivers also varied. Caregiver locations included remote clinics, homes, schools, community centers, conference rooms, and mobile tele-vehicles. Studies including remote clinics were described as healthcare sites that the caregiver attended in-person, where the provider was not physically present, to facilitate virtual appointments. In some remote clinics, the provider and family were co-located within the same clinic, whereas other studies described the provider in a separate location. One study used a mobile tele-vehicle, a vehicle equipped with virtual care equipment, to conduct diagnostic Auditory Brainstem Response in a remote location. Additionally, this study used two support personnel: a village health worker to prepare children for onsite testing and a remote technician who set-up equipment and participated in a videoconference with the audiologist who remotely controlled the Auditory Brainstem Response software.^53^

The included studies varied by the type of intervention delivered to the child, caregiver participation type, caregiver activities, disability/disorder, number of participants, type of caregiver, and the age of the children (Table 1). Most of the children receiving care were preschool or school-aged and their participating caregivers were most frequently between the ages of 25–39 years (*n* = 27). Caregiver participation types were categorized as (a) *attended* or (b) *involved*. *Attendance* referred to observers who were not directly involved in the provision of the intervention (e.g., they were present and/or asked questions to the provider). Caregivers who were *involved* were further categorized as either *assistant*s or *co-providers*. Caregivers categorized as *assistants* were described as providing physical, gestural, and/or verbal cues during the appointment. Those who were *co-providers* were active participants that co-delivered the intervention, sharing responsibilities in virtual delivery while receiving guidance, coaching, and/or feedback from the provider during the process.

**Table 1.** Summary of participant demographics, type of intervention, and caregiver participation type and activities in paediatric speech, language, and hearing intervention studies.

Citation	Type of intervention delivered to the child	Caregiver participation type	Caregiver activities	Disability/disorder (children)	Participants (caregiver (*n*)/total (*N*))	Age of children (years)
Aggarwal et al., 2015	Communication development therapy	Involved: co-provider	Received coaching; co-delivered intervention; interacted with child; intervention feedback to provider	Autism	(2/4) Parent: mother	3–4
Baharav & Reiser, 2010	Speech language therapy	Involved: co-provider	Received coaching; co-delivered intervention; interacted with child; intervention feedback to provider	Autism	(2/4) Parent	4–5
Barbosa et al., 2017	Communication development therapy	Involved: co-provider	Co-delivered intervention; interacted with child; intervention feedback to provider	Autism	(40/80) Parent/caregiver	6–17
Behl et al., 2017	Language development therapy	Involved: co-provider	Received coaching; co-delivered intervention; interacted with child; intervention feedback to provider	Hearing loss	(48/88)^a^ Caregiver/family	19–20 months
Blaiser et al., 2013	Early intervention services	Involved: co-provider	Received coaching; co-delivered intervention; interacted with child; intervention feedback to provider	Hearing loss and: cochlear implant; multiple disabilities; or Trisomy 21^b^	(27/63) Family	10- 28 months
Bridgman et al., 2016	Stuttering therapy	Involved: co-provider	Interacted with child; co-delivered intervention at home after planning & demonstrating procedure with provider; measured stuttering severity	Stuttering	(49/98)^a^ Parent	3–5
Campbell et al., 2021	Speech language therapy	Involved: assistant	Interacted with child providing physical, gestural, & verbal cues; presenting stimulus & task instructions; intervention feedback to provider	Speech and/or language difficulties	(17/26) Parent	3–6
Chen & Liu, 2017	Auditory Verbal Therapy	Involved: co-provider	Received coaching; co-delivered intervention; interacted with child; intervention feedback to provider	Hearing loss; Cochlear implant^b^	(5/10) Parent	5^c^
Ciccia et al., 2011	Language assessment; hearing assessment	Attended: observed	Intervention feedback to provider	Potential hearing or speech difficulties	(160/570) Family	0–6
Constantinescu, 2012	Auditory Verbal Therapy	Involved: co-provider	Received coaching; co-delivered intervention; interacted with child; intervention feedback to provider	Hearing loss	(13/26) Family	3 months – 6 years
Daczewitz et al., 2020	Parent- implemented communication strategies	Involved: co-provider	Received coaching; co-delivered intervention; interacted with child; intervention feedback to provider	Hearing loss	(1/2) Parent: father	26 months
Diez-Juan et al., 2014	Language intervention	Involved: co-provider	Received coaching; co-delivered intervention; interacted with child; intervention feedback to provider	Fragile X full mutation & autism, Developmental Language Disorder; Attention-deficit/hyperactivity disorder; global developmental delay; selective mutism, anxiety disorder & borderline intellectual functioning^b^	(4/8) Parent	2–11
Dharmar et al., 2016	Hearing assessment	Attended: observed	Intervention feedback to provider	Did not pass hearing screen	(11/33) Parent	5.5^c^ months
Fairweather et al., 2016	Therapy (anxiety, verbal conflicts, speech & concentration)	Attended: observed	Intervention feedback to provider	Speech and language difficulties	(6/12) Parent/caregiver	7–15
Fordington & Brown, 2020	Hearing assessment	Attended: observed	Intervention feedback to provider	Suspected/diagnosed otitis media with effusion. Trisomy 21; learning disability^b^	(60/120) Caregiver	2–8
Fuller & McLeod, 2019	Music therapy	Involved: assistant	Interacted with child; intervention feedback to provider	Hearing impairment	(27/56) Parent/grandparent	2–5
Goehring & Hughes, 2017	Cochlear implant fitting	Attended: observed	Intervention feedback to provider	Hearing loss cochlear implant users	(19/38) Parent/caregiver	2–7
Hatton et al., 2019	Hearing assessment (auditory brainstem response)	Attended: observed	Intervention feedback to provider	Potential hearing loss	(41/143) Caregiver	2.3^c^ months
Havenga et al., 2017	Early intervention services	Involved: co-provider	Interacted with child; co-delivered intervention; intervention feedback to provider	Hearing loss	(10^a^/20) Parent: mother	1–5
Hines et al., 2019	Speech language therapy	Attended: observed	Intervention feedback to provider	Autism	(4^d^/8) Parent: mother	5–8
Hughes et al., 2018	Cochlear implant fitting	Attended: observed	Intervention feedback to provider	Hearing loss cochlear implant users	(17/34) Caregiver	1–3
Isaki & Farrell, 2015	Speech language therapy	Involved: assistant	Assisted child with technology	Apraxia of speech & cognitive delay; apraxia of speech; cleft lip & palate; developmental articulation disorder^b^	(5/10) Parent	4–9
Manning et al., 2020	Language assessment	Involved: assistant	Interacted with child	Not reported	(62/124) Family	1–3
M. McCarthy et al., 2020	Early intervention services	Attended: observed	Provided feedback on intervention	Hearing loss Multiple disabilities^b^	(141/141) Parent: mother, father	2 months – 8 years
McDuffie et al., 2018	Naturalistic parent-implemented language intervention	Involved: co-provider	Received coaching; co-delivered intervention; interacted with child; intervention feedback to provider	Fragile X syndrome	(20/20) Parent: mother	10–16
McDuffie et al., 2016	Naturalistic parent-implemented language intervention	Involved: co-provider	Received coaching; co-delivered intervention; interacted with child; intervention feedback to provider	Fragile X syndrome	(6/12) Parent: mother	2–6
Muñoz et al., 2017	Hearing aid management	Involved: assistant	Interacted with the child using behavior management strategies; intervention feedback to provider	Hearing loss	(4/8) Family, mother, grandmother	Birth – 5
Muñoz et al., 2021	Hearing aid management	Attended: observed	Received eHealth programmes providing hearing aid management education; intervention feedback to the provider	Hearing loss, Behind-the-Ear hearing aid only	(82/164) Parent: mother, father	Birth – 3
O’Brian et al., 2014	Language development therapy	Involved: co-provider	Interacted with child; co-delivered intervention at home; measured stuttering severity; intervention feedback to provider	Stuttering	(3/6) Family	3–4
Pamplona & Ysunza, 2020	Speech language therapy	Involved: assistant	Interacted with the child during activities & reinforced articulation	Cleft palate	(43^a^/86) Family	4–12
Parnandi et al., 2013	Speech language therapy	Attended: observed	Assisted child by demonstrating activity; intervention feedback to the provider	Apraxia of speech	(3/7) Parent	3–7
Parnandi et al., 2015	Speech language therapy	Attended: observed	Assisted child by demonstrating activity; intervention feedback to provider	Apraxia of speech	(7/15) Parent	4–10
Pennington et al., 2019	Speech & language development therapy	Attended: observed	Intervention feedback to provider	Cerebral palsy & dysarthria	(29/51) Parent	8.8^c^
Quinn et al., 2021	Language development therapy	Involved: co-provider	Received coaching; co-delivered intervention; interacted with child; intervention feedback to provider	Developmental delay & premature; language impairment; Trisomy 21^b^	(4/8) Parent	1–2
Raatz, Ward, Marshall, & Burns, 2021	Paediatric feeding assessments	Attended: observed	Intervention feedback to provider	Feeding disorders 60% had >1 area of feeding difficulties	(40/80) Parent	4 months – 7 years
Raatz, Ward, Marshall, Burns et al., 2021	Paediatric feeding assessments	Attended: observed	Intervention feedback to provider	Feeding disorders	(44/44) Parent	Birth – 7
Ramkumar et al., 2016	Hearing assessment; Remote diagnostic	Attended: observed	Intervention feedback to provider	Potential hearing loss	(87/206) Parent: mother	Not stated
Saul & Norbury, 2020	Speech language therapy	Attended: observed	Intervention feedback to provider	Verbally autistic <10 sounds, 20 words & <5 words at visit	(19/38) Parent	3–6
Sicotte et al., 2003	Stuttering therapy	Attended: observed	Intervention feedback to provider	Stuttering & difficulty with 5% of syllables	(6^a^/12) Parent	3–19
Song et al., 2016	Parent-child communication	Involved: co-provider	Received coaching; co-delivered intervention; interacted with child; intervention feedback to provider	Language delays	(8/16) Caregiver	2–3
Steuerwald et al., 2018	Hearing aid/Cochlear implant management & programming	Attended: observed	Assisted child	Trisomy 21 & hearing loss	(2^b^/10) Parent: mother	12
Stith et al., 2012	Listening and spoken language therapy	Involved: co-provider	Co-delivered intervention; interacted with child	Hearing loss; cochlear implant^b^	(2/4) Parent	2–3
Sutherland et al., 2017	Language assessment	Attended: observed	Intervention feedback to provider	Reading difficulty & known or suspected language impairment	(23/49) Parent	8–12
Tanner et al., 2020	Therapy	Attended: observed	Intervention feedback to provider	Not recorded	Unknown Caregiver^e^	Not stated
Thomas et al., 2016	Language intervention	Attended: observed	Intervention feedback to provider	Apraxia of speech	(5/15) Parent	5–11
Thomas et al., 2017	Language intervention	Involved: co-provider	Delivered intervention; intervention feedback to provider	Apraxia of speech	(10/10) Parent: mother, father	5–11
Valentine, 2015	Stuttering therapy; language development therapy	Attended: observed	Intervention feedback to provider	Stuttering	(2/4) Caregiver	11
Whitehead et al., 2012	Language assessment	Attended: observed	Intervention feedback to provider	Surgical cleft palate repair	(9/18) Parent	5–14

^a^ Estimate based on a 1-on-1 child-to-caregiver ratio.

^b^ Indicates some of the children in the study had a combination of diagnoses, not limited to any one diagnosis.

^c^ Indicates an average age of child.

^d^ Indicates only results for one child-caregiver pair was analyzed based on inclusion/exclusion criteria.

^e^ The number of total respondents was reported, no details were provided on the number of included caregivers.

### Categories influencing caregiver participation in virtual care

Inductive content analysis revealed eight main categories and 24 sub-categories relating to barriers and facilitators to virtual caregiver participation (Table 2). The main categories related to *attitudes*, *behavioral considerations* for the child(ren), *environment*, *opportunities*, *provider-family relationship*, *roles in the care process*, *support*, and *technology*. Many studies cited more than one facilitator and/or barrier, resulting in a total of 299 facilitators and 118 barriers. Across all included studies, the facilitators to participation were most often reported as having opportunities to participate (*n* = 78), followed by technology-related factors (*n* = 68); whereas the barriers to caregiver participation were most often associated with technological challenges (*n* = 48), followed by the environment (*n* = 13), and the role(s) of the caregiver and child in the virtual care process (*n* = 14).

**Table 2.** Categories influencing caregiver participation in virtual care.

Category	Subcategories	Element	Total (N)	Facilitator (*n*)	Barrier (*n*)
Attitudes			*65*	*57*	*8*
	Caregiver attitude	Confidence in quality of care		1	0
		Motivation		2	0
		Understands participation benefits		1	0
		Virtual care experience		5	0
		Virtual care satisfaction		17	0
		Willingness to use/recommend		14	1
	Child attitude	Towards application		3	0
		Towards interactive & motivating nature of technology		5	0
		Virtual care as positive experience		2	0
	Delivery model preference	Caregiver preference		7	7
Child behavioral considerations			*12*	*3*	*9*
		Attention impairments/tantrum		0	3
		Difficulty sitting in same place		0	2
		Emotional regulation		0	1
		Social interactions		1	1
		Technology impact on anxiety		2	2
Environment			*31*	*18*	*13*
	Contextual factors	Level of environmental distraction		2	4
		Physical space		1	2
		Procuring physical objects		0	1
		Reduces stress from travelling		1	1
		Treatment normalized in home environment		1	1
		Value of home environment		7	2
	Family involvement	Convenience of family participation		1	0
		Family background		0	1
		Other family member participation		5	1
Opportunities			*88*	*78*	*10*
	Accessibility	Accessibility of virtual care		7	0
		Impact to wait-time		3	0
	Attendance/scheduling	Cancellations/absences		4	2
		Scheduling		7	1
	Cost	Cost-benefit/fiscal savings		16	0
	Convenience	Convenience of virtual care		12	0
		Out-of-pocket payment		0	1
	Time	Comparison to in-person		9	1
		Lack of time for virtual care		0	1
		Resource preparation time		0	1
		Time off work		2	1
		Time spent fixing technical issues		0	1
		Travel time		18	1
Provider-family relationship			*18*	*10*	*8*
	Communication	Communication difficulties		0	4
		Conversation with provider		1	1
		Increased contact/sessions with provider		2	0
		Provider engagement with younger children		0	1
		Provider responsiveness		1	0
	Rapport	Collaborative relationships		1	0
		Rapport building		4	2
Roles in care process			*37*	*23*	*14*
	Caregiver role	Active participant		5	0
		Engagement/involvement		6	0
		Manage behavioral problems		0	1
		Reduced pressure for caregivers who have many duties		2	0
		Role extended		1	3
	Child engagement	Engagement		4	9
		Participation		2	1
		Promotes child independence		3	0
Support			*49*	*42*	*7*
	Support personnel	Required IT expert		0	1
		Trained and non-trained personnel		13	0
	Training	Caregiver training		13	0
	Prior experience	Caregiver device experience (computer, tablet/iPad, phone)		4	0
		Caregiver videoconferencing experience		5	0
		Caregiver virtual care experience		3	5
		Child technology experience		4	1
Technology			*116*	*68*	*48*
	Access	Cost of equipment		0	1
		Provision of technology		24	0
		Technology as a participation requirement		0	11
	Caregiver aptitude	Proficiency with technology		3	3
	Comfort	Caregiver comfort with technology		21	4
		Child comfort with technology		4	1
	Privacy/security	Appointment recordings		1	1
		Privacy respected		1	0
	Quality	Technology quality		8	1
	Usage	Increase in child's screen time		0	1
		Innovative use of technology		3	1
		Ease of use		3	24
		Integration		0	1

#### Attitudes

The category *attitudes* is defined as a general and enduring positive or negative feeling about virtual care.^54^ The sub-categories that affect *attitudes* include how the caregiver and/or child feels about virtual care (general attitudes of a person) and delivery model preference (e.g., phone versus videoconferencing). Attitudes were frequently reported across studies to act as a facilitator to caregiver participation. While a range of attitudes towards virtual care were reported, generally caregivers and children reported positive attitudes. In some cases, caregivers were more empowered and engaged in virtual care when compared to traditional in-person care.^55,56^ Willingness to participate was reported to be associated with feelings of frustration towards technology as difficulties were encountered.^57,58^ Caregiver delivery model preference was mixed, some caregivers preferred virtual care, some had preferences for hybrid models, and others preferred in-person services.^53,59–66^

#### Child behavioral considerations

This category includes considerations made based on how the child conducts themselves (actions, reactions, and functioning) in response to the virtual care environment, including challenging behaviors that may interfere with the caregivers’ participation in virtual care. The *child behavioral considerations* category was the least reported category and was often reported as a barrier to caregiver participation. In scenarios where the child(ren) experienced tantrums, were less emotionally regulated, or had difficulties staying in the same position for some duration of time, the caregiver's ability to participate was negatively affected.^52,67–71^ Furthermore, the child's comfort with technology and with their home environment was reported to reduce anxieties associated with participating in appointments.^72,73^

#### Environment

The *environment* was defined as the physical and social environments in which the virtual care sessions were delivered that acts as either a barrier or facilitator to the person's functioning.^74^ The most cited benefit to caregiver participation was related to the perceived value of the home environment.^61,69,73,75–78^ Virtual care allowed providers the opportunity to observe naturalistic interactions between caregivers and their child, without being intrusive.^79^ One study noted the potential for virtual care provided at home to help normalize the care process as it is a familiar and comfortable environment for the child.^80^ The ability to include other family members in the virtual care process was a reported facilitator for caregiver participation.^52,60,72,76,81^ In cases where the home was distraction-free, this enabled care; however, when distractions were present this was reportedly a significant environmental barrier to care.^66,69,71,82^ Only one study specifically identified the influence a family's background may have on their participation in virtual care; this study reported that caregivers from disadvantaged backgrounds had lower levels of self-efficacy and involvement in virtual care.^83^

#### Opportunities

This category is defined as the set of physical and social circumstances that make the virtual delivery of care more possible or desirable and is comprised of the following sub-categories: *accessibility*, *attendance and scheduling*, *convenience*, *cost*, and *time*.^84^ This category and its associated sub-categories were most frequently cited as facilitators to caregiver participation. Caregivers frequently reported that they valued the reduction in travel time and overall disruption to their family routine offered by virtual appointments. Additionally, many studies reported being able to better schedule virtual appointments around their work schedules.^53,60,61,64,66,69,71,73,76,78,80,85–91^ In a few studies, families reported receiving care in a timelier manner when compared to traditional in-person care.^53,73,85^ Overall, reduced travel needs, lower care-related costs, and the general convenience of attending virtual appointments were reported as the top three facilitators to caregiver participation. *Opportunity*-related barriers were related to scheduling difficulties and time constraints. The sub-category of *attendance* often related to scheduling difficulties, technology challenges, and poor family attendance.^60,77,82^ Caregivers reported a perceived increase in time for the virtual appointment relating to their need to organize and/or prepare resources to use during the appointment (e.g., cutting cue cards before the session).^52,61,67,77^ In addition, appointment length was reported to increase when additional time was needed to troubleshoot technical difficulties.^75^

#### Provider-family relationship

This category was defined as a therapeutic relationship that centers around good communication, patient trust and connection, a shared understanding of goals, and access to care.^92,93^ In two cases, providers described satisfaction with the quality of communication during the session.^70,94^ Some caregivers reported feeling comfortable communicating with providers when engaging in virtual care.^59,60,71,75,88,95^ In addition, caregivers noted that when compared to in-person appointments, virtual care offered improvements in conversation and more frequent contact with their providers.^71,91,94^ Turn-taking during group discussions was cited as an important consideration for group-based interventions.^94^ Communication challenges were related to difficulty understanding provider explanations, provider difficulty communicating with families when English was the family's second language, and a general lack of communication.^53,61,72^ These communication difficulties acted as barriers to building rapport.^72,91^

#### Role in the care process

This category includes child and caregiver engagement in the virtual care process, which can change over time. At a minimum, all caregivers in this review participated in a passive role, meeting the criteria of attendance. Caregivers who participated in appointments beyond only attending were categorized as being involved in the intervention and held roles on the continuum between passive (involved but limited decision-making authority) and active participation, such as assisting the child to facilitate the intervention and co-delivering the intervention (shared responsibility). Co-providing caregivers typically had a high level of participation and significant associated activities; this included the use of coaching interventions to support successful virtual care participation. This review identified an inverse relationship with child age and caregiver role: as the child's age increased into adolescence, caregiver roles became more passive. The exception to this role reduction involved adolescent children with complex healthcare needs, as they often relied on caregivers to take on more active roles.

Some of the caregivers reported feeling more engaged and involved in their child's care.^77,87,88,96^ Other caregivers thought their roles were extended during virtual care and sometimes these new roles added increased stress.^67,68^ One study reported that caregivers felt uncomfortable in co-provider roles in situations where they had to give their child negative feedback.^71^ Child engagement was infrequently reported in the included studies; however, when engagement was reported, many cited difficulties related to keeping the child engaged.^52,63,64,71,78,87,97–99^ Another study reported that the child was unwilling to participate in virtual intervention.^72^ In contrast, some studies cited that virtual care promoted the child's independence, engaged the child, and motivated them to participate.^59,63,68,69,75,99–101^

#### Support

Training, prior experience, and support personnel were sub-categories within the *support* category. This category includes the personnel and physical assistance that caregivers received during, or prior to, the delivery of virtual care that can aid in successful participation. Some studies reported the provision of formal training to caregivers prior to participation in a virtual appointment (*n* = 13), however, there was minimal information reported by these studies to describe the training elements and procedures. In the studies that did provide information, four studies reported the provision of general technology training and an additional study reported functional training related to screen positioning.^71,73,75,89,94^ Training specific to information technology required for auditory-verbal therapy, as well as non-specific technology to assist with an internet connection was provided in two studies.^64,81^ Some studies provided caregivers with technology-specific training, including videoconferencing applications (*n* = 3), tablet/smartphone applications (*n* = 2), operation of iPads/tablets (*n* = 3), or computers (*n* = 1), and the Kubi robot (a stand for tablets acting as a ‘robot’ allowing the provider to move around and tilt the tablet as if they were in-person; (*n* = 1).^57,68,81,88,94,97,102^ Some studies reported on previous experience, ranging from no experience, device-specific experience, videoconferencing experience, and, more broadly, virtual care experience.^61,66,69,73,77,80,85,88–90,94,97^

Support personnel performed a variety of tasks under the direction of the provider and were used at a variety of locations. Support personnel were required when hands-on components and/or the use of specialized equipment were required to provide services. This support included the physical placement of electrodes or probes for auditory brainstem response testing, connecting programming cables, positioning otoscopes, conducting distortion product otoacoustic emission screening, and tympanometry.^53,62,65,85,96^ Trained support personnel (e.g., information technology support staff, other health care professionals, or clinical students) were used to facilitate assessments and/or interventions by evaluating feeding skills, using toys to capture the child's attention, modelling actions for families, and engaging the child in behavioral tasks.^59,61,72,98,103,104^ Some families required support personnel to aid in the set-up and/or use of technology.^76,97,105^ In addition to the caregiver, family members could act as support personnel and may have received training prior to virtual care.

#### Technology

**Figure 2.**
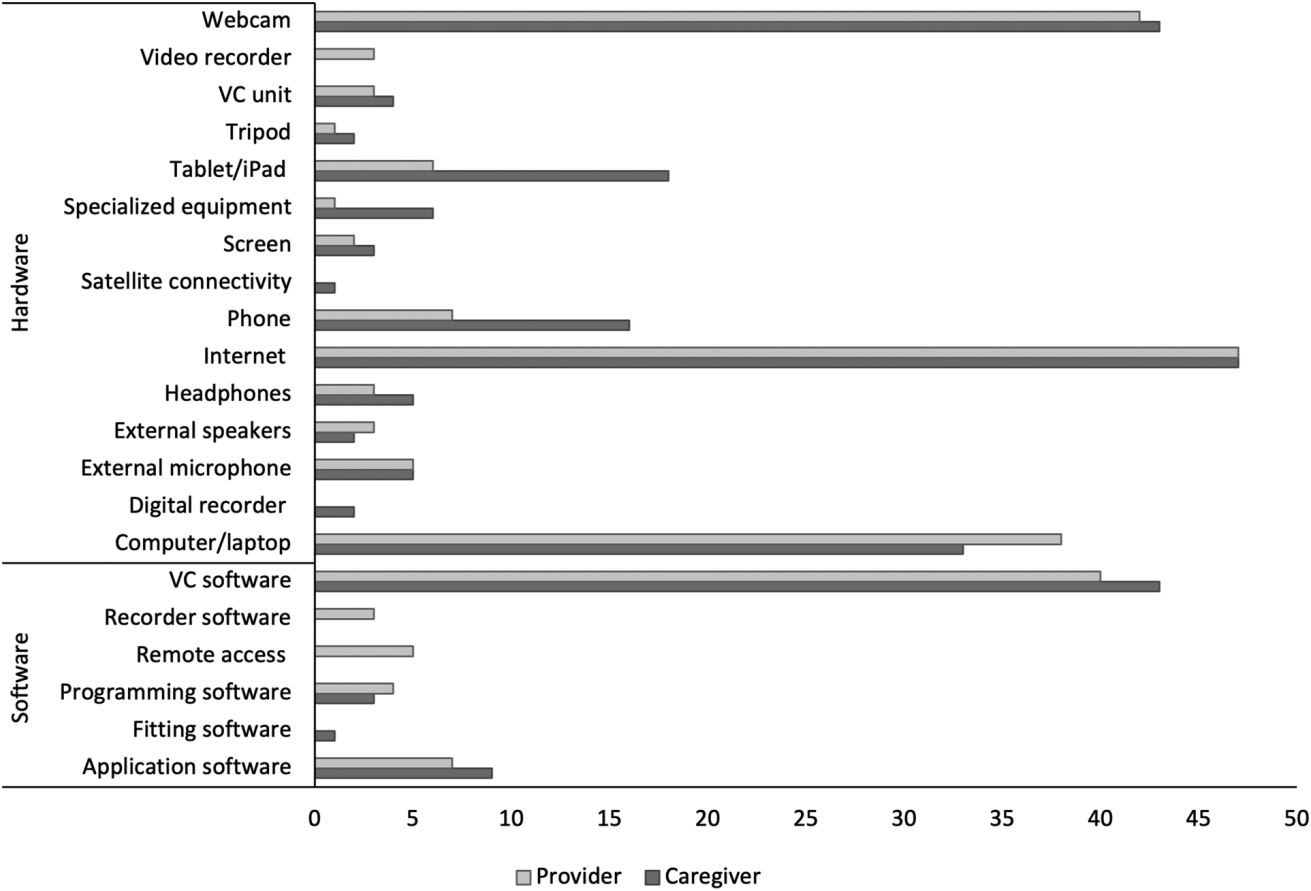
Technologies used in virtual appointments.

This category is defined as any equipment or software used in the delivery of virtual care services; this may be technology used in the home or from a remote location.^106^ Each study had unique technology and equipment needs which varied according to whether the user was a provider or a caregiver (e.g., remote location with client) and were specific to the type of location (Figure 2).

#### Note

VC stands for videoconferencing and phone included the use of smartphones.

Included within the technology category were the following sub-categories: *access*, *caregiver aptitude*, *comfort*, *privacy/security*, *quality*, and *usage*. All provider and caregiver locations incorporated the internet, with the majority using webcams and videoconferencing software to facilitate virtual appointments. In the case of asynchronous delivery, webcams and videoconferencing were not used. The tele-vehicle study incorporated internet access through satellite connectivity.^53^ In general, remote clinics had more standardized technology and equipment requirements, while home locations had the greatest variety of requirements. These home-based requirements often varied based on the type of intervention being provided.

In this review, access to technology was the highest reported facilitator to participation. Caregivers either had access to their own technology or were provided with technology in their remote location. Most of the studies that reported on caregivers in a remote location other than their home, mentioned sites such as schools, mobile tele-vehicles, remote clinics, community rooms, or conference rooms. In these studies, the necessary technologies were provided on-site to facilitate the virtual appointments and included internet access (*n* = 11), videoconferencing software (*n* = 9), webcams (*n* *=* 9), computers (*n* = 4), specialized equipment (*n* = 4), tablets (*n* = 3), videoconferencing units *(n* = 3), application software (*n* = 2), programming software (*n* = 2), an external microphone (*n* = 1), and satellite connectivity in lieu of Wi-Fi (*n* = 1).^53,58,59,62,63,65,70,82,85,96–99^ Studies where the caregivers received virtual care at their home provided fewer details around technology access. Under half of the studies had inclusion criteria that required caregivers to have access to technologies to participate, which included access to reliable internet (*n* = 12), devices with videoconferencing capabilities (*n* = 5), and webcams (*n* = 2).^61,66,69,73,76,80,89,95,103,107,108^ To ensure access to reliable internet, one study had caregivers conduct an online internet speed-test the day before their appointment at the same time as the scheduled appointment.^72^

*Ease of use* was the most cited technology barrier and the most cited overall barrier to participation. These barriers were generally related to audio and/or visual quality, volume, and internet problems; however, they were inconsistently reported across studies. Internet problems were reported to be associated with connectivity issues, bandwidth, and internet speed. Reported technical difficulties related to audio quality included distortion, audio delays, echoes, low volume, and the addition of background noise.^53,58,61,66,70–72,75,82,86,94,97,98,102,107^ One study reported that the provider switched from a virtual appointment to a phone-based appointment due to poor audio quality.^75^ Two SLP studies identified that audio quality problems could affect the ability to understand a child's speech sounds, making service delivery challenging.^86,102^ Providers had trouble assisting families in regulating computer volume over videoconferencing, as caregivers were unsure if their volume could be adjusted or if a particular volume level was required during the intervention.^94^

Poor visual quality was often reported as freezing, missing, or of low quality.^57,63,71,72,75,82,97^ One study associated decreased audio/video quality and connectivity problems with the time of day when internet usage in the community increased.^66^ Reported equipment difficulties included webcams, software, and/or other applications. Technology and the positioning of technology were identified as elements that may impact communication during virtual care.^60,65,75,97,102,109^ The stationary positioning of webcams and the participants’ ability to stay within the visible range often made provider observations difficult. Some providers hesitated in requesting adjustments to the webcam to avoid interruptions to the naturalistic flow of the appointment.^94,110^ Technical problems resulting from application software, such as failing to log in, long loading periods, and software crashes, resulted in feelings of frustration from the families involved.^57,63^ Connectivity issues related to videoconference applications skipping or crashing were also reported by a handful of studies.^53,69,76,89,97^

Technical quality was a facilitator to caregiver participation in eight studies and was often related to caregivers experiencing good audio/visual quality.^53,58,62,64,73,95,96,98^ Technology comfort was more often reported as a facilitator, with caregivers feeling comfortable with technology. Caregivers’ comfort with technology was reported as a barrier to participation in some studies, specifically when caregivers were worried about connecting hearing aids to computers.^66,77,81^ Related to this concern, one study reported a family who discontinued virtual appointments under the perception that the burden of learning a new technological set-up was too difficult.^87^

## Discussion

This scoping review gathered, synthesized, and summarized peer-review literature from 48 studies within the field of audiology and/or SLP that included the virtual delivery of paediatric assessments and/or interventions with caregiver participation. This review identified barriers and facilitators reported to influence caregiver participation in virtual audiology and SLP care. Eight categories emerged from the review, including *technology*, *support*, *opportunities*, *attitudes*, *role in the care process*, *environment*, *child behavior*, and *provider-family relationship*. These categories are related to both barriers and facilitators. In addition, this review identified novel findings related to the delivery models commonly used with caregivers, technological/equipment requirements, and levels of caregiver participation.

### Virtual care delivery models

Synchronous videoconferencing was the most frequently cited delivery method, either on its own or as part of a hybrid model. This aligns with current speech, language, and hearing research, indicating a preference or bias for synchronous videoconferencing methods when providing virtual care.^111,112^ Synchronous videoconferencing is considered the gold standard for virtual care as it most closely approximates in-person care by providing face-to-face elements, real-time clinical decisions, non-verbal cue reading, and offers a glimpse into the family's home environment.^3,44^ In addition, the provision of visual cues to assess communication ability is important for care providers to assess, and can supplement poor audio cues, for example, when participants have a hearing impairment.

Hybrid delivery of care allowed providers to combine different models of care to meet the needs of the appointment and/or family. Existing research on family-centered paediatric telehealth suggests that a hybrid model (e.g., the combination of virtual services paired with in-person), will likely become the standard care delivery model.^44^ By tailoring virtual care delivery models and modalities to meet the needs of each family, the provider can foster family-centered care. The flexibility of virtual and hybrid delivery models has the potential to create opportunities for caregivers to become part of the decision-making process and facilitates participation from multiple family members.^52,60,72,76,81^ The type of delivery model used will influence the technology requirements, training, and support personnel needs of each client; therefore, it is critical that professionals consider the appropriateness of the delivery model for each family.

### Technology and equipment requirements

Technology was most often reported to act as a barrier to caregiver participation, including problems relating to connectivity and internet, software, and audio and/or visual quality, consistent with existing literature.^55,113,114^ Preventative measures aimed at mitigating technology difficulties, such as pre-appointment internet speed-tests or pre-established alternative technology plans, may be implemented by providers and/or organizations to improve virtual participation experiences. The most frequently reported facilitator to participation in this review was the sub-category access. Caregivers in some studies were provided access to the necessary technologies needed for their child's virtual care appointment, thus facilitating their participation. The ‘digital divide’, referring to the unequal access to digital technologies, often plays a role in the success and integration of virtual care services.^115,116^ While many of the caregivers in the included studies were provided technology at their remote location, providers must consider how technology access can be improved outside of research contexts. Providers can consider offering loaner equipment and/or accessing/initiating technology donations as a means of increasing technology access for families. Having access, alone, is not sufficient to ensure caregiver participation, however, attitudes and aptitudes of the family receiving care should be investigated to determine if the family is a good candidate for a virtual care delivery model.

If virtual service delivery is to become ‘the new normal’ in virtual speech, language, and hearing care, it is imperative that caregivers receive appropriate training and resources before appointments to reduce potential caregiver dropout and/or technology or participation barriers. Recent literature has identified the need for virtual care training for clinicians and caregivers; one study reported caregivers have a desire to receive virtual care training.^117–119^ Technology training can increase caregiver comfort and aptitude with technology and reduce negative attitudes towards virtual care resulting from technological difficulties. These findings identify the need for training modules and/or candidacy assessments in audiology and SLP to address the technology components of virtual care so that providers and caregivers can feel confident when selecting virtual care as a delivery method.

### Caregiver participation roles

Caregiver participation roles were found to vary within the broader classifications of *attending* and *involving*. Everyone involved in the virtual care processes has unique roles, including the caregivers, children, and care providers. Roles vary depending on the type of care and the context of the virtual appointment. The provider's participation role was not investigated as an objective of this review. This study identified that caregivers are often required to take on more active participation roles, especially for younger children, during virtual care.

Consistent with existing literature, caregivers of younger children are often highly involved in the process and required to fulfil multiple roles to facilitate the participation of their child in virtual appointments.^117^ Although infrequently reported, caregiver participation can be influenced by their child's temperament, specifically when challenges arise resulting from the child's behavior and attention.^25,120,121^ Strategies and training for handling children's behavioral situations might be useful tools for providers and caregivers to refer to in virtual intervention contexts.^68,122^

Caregivers were noted to have active roles as assistants, facilitators, or co-therapists during their child's virtual appointments. Active caregiver participation and home-based delivery of care seem to provide appropriate circumstances for the application of therapy goals and skills development in the home environment. However, home-based virtual care may be negatively impacted by distractions (i.e., other responsibilities of caregivers such as work or other children). Prior to engaging in virtual appointments, caregivers can be instructed to reduce distractions in their background by removing clutter from their visible workspace and eliminate extraneous noise, for example, pets and/or people.^123,124^ Providers will also need to adjust their treatment to be delivered across a virtual modality with the loss of physical touch elements. Support personnel may be integrated into the care process to assist with challenging tasks, provide physical components of care, and/or reduce caregiver demands during virtual appointments.^3,31,125^ The findings from this review suggest effective communication between the provider and the caregiver is necessary to establish and maintain rapport and facilitate a therapeutic relationship.

### Limitations and future directions

This review has some limitations. By limiting the literature search to peer-reviewed articles written in English, it is possible that potentially relevant information from other sources may have been missed. A second limitation relates to the location of the studies included. As most studies included in this review were conducted in the Global North, findings from this review may not reflect virtual care experiences across all contexts with different cultures and where access to technology, connectivity, and digital literacy may be reduced considerably. While some studies included the Global South, more research is needed to explore the experience of virtual care in these geographic areas. It is impossible for literature to keep up with the ever-evolving state of technology and innovations, therefore, this review should be periodically replicated to ensure new evidence is added.

Caregiver participation in virtual care across the included studies may reflect the experience(s) of people who have access to specific resources, including internet and computers and not be reflective of a more diverse population. More research is needed to understand the impact of caregiver socio-demographics on participation in virtual audiology and SLP care, necessitating increased reporting on socio-demographic characteristics (i.e., age, gender, education, and employment) of the individuals participating in virtual care.^121,126^ The studies included in this review were mainly follow-up appointments, the appointment type prior to the follow-up study was not identified as part of this review. Future research should investigate if patterns exist for caregiver participation related to the type of delivery model and time point at which care was delivered such as initial or follow-up and hybrid care (in-person and virtual) compared to caregivers who are only offered virtual services.

Future research should be undertaken to improve knowledge related to caregiver technology aptitude, the effect of prior experiences, and training for virtual interventions. These areas were not widely discussed in the studies included in this review and therefore there is a need to investigate their effects on caregiver participation. In addition, an investigation into which areas of training for virtual care would be most beneficial for caregivers is needed. Researchers should be encouraged to outline resources and training materials offered for caregivers during the virtual care process, including manufacturer-created instructions, such as the virtual care platform (e.g., Zoom) and/or device (e.g., Apple iPad). Future research should explore the impact of a caregiver's readiness for virtual care on their participation in virtual care interventions.

## Conclusions

The findings of this scoping review revealed important categories that may influence the participation of caregivers in speech, language, and/or hearing assessment and intervention appointments, especially for young children. Furthermore, specific barriers/facilitators to caregiver participation were identified that might provide guidance for providers and enable identification of a priori needs and supports leading to greater involvement in virtual care. The resultant identification of eight main categories and 24 sub-categories relating to barriers and facilitators to virtual caregiver participation can be used to assist in the development of models and clinical tools that can contribute to standards for flexible, family-centered virtual care practice initiatives.

## Supplemental Material

sj-docx-1-dhj-10.1177_20552076231216684 - Supplemental material for Barriers and facilitators to paediatric caregivers’ participation in virtual speech, language, and hearing services: A scoping review Click here for additional data file. Supplemental material, sj-docx-1-dhj-10.1177_20552076231216684 for Barriers and facilitators to paediatric caregivers’ participation in virtual speech, language, and hearing services: A scoping review by Danielle DiFabio, Sheila Moodie, Robin O’Hagan, Simrin Pardal and Danielle Glista in DIGITAL HEALTH

sj-docx-2-dhj-10.1177_20552076231216684 - Supplemental material for Barriers and facilitators to paediatric caregivers’ participation in virtual speech, language, and hearing services: A scoping review Click here for additional data file. Supplemental material, sj-docx-2-dhj-10.1177_20552076231216684 for Barriers and facilitators to paediatric caregivers’ participation in virtual speech, language, and hearing services: A scoping review by Danielle DiFabio, Sheila Moodie, Robin O’Hagan, Simrin Pardal and Danielle Glista in DIGITAL HEALTH

## Appendix A Ovid MEDLINE Search Strategy

1. telemedicine.mp. or exp Telemedicine/
2. remote.mp.
3. “remote consultation”.mp.
4. exp Remote Consultation/
5. virtual.mp.
6. internet.mp.
7. exp Internet/
8. “internet-based”.mp.
9. "internet based”.mp.
10. “tele-medicine”.mp.
11. “tele-audiology”.mp.
12. “teleaudiology”.mp.
13. “tele-health”.mp.
14. telehealth.mp.
15. exp Mobile Applications/
16. “mobile applications”.mp.
17. Mobile.mp.
18. Ehealth.mp.
19. mhealth.mp.
20. “e-health”.mp.
21. connected.mp.
22. cyber.mp.
23. online.mp.
24. digital.mp.
25. “digital technology”.mp.
26. exp Digital Technology/
27. videoconferencing.mp. or exp Videoconferencing/
28. videorecording.mp. or exp Video Recording/
29. ‘video recording”.mp.
30. “video based”.mp.
31. “video-based”.mp.
32. teleconsultation.mp.
33. telerehabilitation.mp. or exp Telerehabilitation/
34. “web based”.mp.
35. “web-based”.mp.
36. telepractice.mp.
37. exp Web Browser/
38. “web browser”.mp.
39. “technology-assisted”.mp.
40. “technology assisted”.mp.
41. telecare.mp.
42. telehearing.mp.
43. “tele-hearing”.mp.
44. teleintervention.mp.
45. “tele-intervention”.mp.
46. 1 or 2 or 3 or 4 or 5 or 6 or 7 or 8 or 9 or 10 or 11 or 12 or 13 or 14 or 15 or 16 or 17 or 18 or 19 or 20 or 21 or 22 or 23 or 24 or 25 or 26 or 27 or 28 or 29 or 30 or 31 or 32 or 33 or 34 or 35 or 36 or 37 or 38 or 39 or 40 or 41 or 42 or 43 or 44 or 45
47. counselling.mp. or exp Counseling/
48. “health screening”.mp.
49. “hearing screening”.mp.
50. evaluation.mp.
51. assessment.mp.
52. service.mp.
53. “early intervention”.mp.
54. exp Early Intervention, Educational/or “early childhood intervention”.mp.
55. “early medical intervention”.mp.
56. “internet based intervention”.mp.
57. “internet-based intervention”.mp.
58. exp Internet-Based Intervention/
59. education.mp. or exp Education/
60. “education of hearing disabled”.mp. or exp “Education of Hearing Disabled”/
61. treatment.mp.
62. “health care”.mp.
63. healthcare.mp.
64. “health-care”.mp.
65. “delivery of health care”.mp. or exp “Delivery of Health Care"/
66. “health service”.mp.
67. therapy.mp.
68. exp Therapeutics/or therapeutics.mp.
69. rehabilitation.mp. or exp Rehabilitation/
70. “health appointment”.mp.
71. “health plan”.mp.
72. “health program”.mp.
73. “health programme”.mp.
74. “health care delivery”.mp.
75. “knowledge translation”.mp.
76. tool.mp.
77. toolkit.mp.
78. “tool kit”.mp.
79. support.mp.
80. training.mp.
81. resources.mp. or exp Health Resources/
82. “health resources”.mp.
83. “resource material”.mp.
84. learning.mp.
85. instruction.mp.
86. (instructional film and video).mp.
87. exp Health Education/or “health education”.mp.
88. “health communication”.mp. or exp Health Communication/
89. 47 or 48 or 49 or 50 or 51 or 52 or 53 or 54 or 55 or 56 or 57 or 58 or 59 or 60 or 61 or 62 or 63 or 64 or 65 or 66 or 67 or 68 or 69 or 70 or 71 or 72 or 73 or 74 or 75 or 76 or 77 or 78 or 79 or 80 or 81 or 82 or 83 or 84 or 85 or 86 or 87 or 88
90. exp Parents/or parents.mp.
91. parent.mp.
92. parenting.mp.
93. parental.mp.
94. “parent-child relations”.mp.
95. exp Parent-Child Relations/
96. mother.mp. or exp Mothers/
97. “mother-child relations”.mp. or exp Mother-Child Relations/
98. mothering.mp.
99. maternal.mp.
100. mom.mp.
101. mothers.mp.
102. fathers.mp. or exp Fathers/
103. “father-child relations”.mp. or exp Father-Child Relations/
104. fathering.mp.
105. father.mp.
106. paternal.mp.
107. dad.mp.
108. caregiver.mp.
109. caregivers.mp. or exp Caregivers/
110. exp Family/or family.mp.
111. “family relations”.mp. or exp Family Relations/
112. families.mp.
113. guardian.mp.
114. guardians.mp.
115. “legal guardian”.mp.
116. exp Legal Guardians/or “guardianship legal”.mp.
117. 90 or 91 or 92 or 93 or 94 or 95 or 96 or 97 or 98 or 99 or 100 or 101 or 102 or 103 or 104 or 105 or 106 or 107 or 108 or 109 or 110 or 111 or 112 or 113 or 114 or 115 or 116
118. child.mp. or exp Child/
119. children.mp.
120. infants.mp. or exp Infant/
121. infant.mp.
122. baby.mp.
123. babies.mp.
124. newborn.mp. or exp Infant, Newborn/
125. pediatric.mp. or exp Pediatrics/
126. pediatrics.mp.
127. toddler.mp.
128. toddlers.mp.
129. paediatric.mp.
130. paediatrics.mp.
131. kid.mp.
132. kids.mp.
133. boy.mp.
134. girl.mp.
135. 118 or 119 or 120 or 121 or 122 or 123 or 124 or 125 or 126 or 127 or 128 or 129 or 130 or 131 or 132 or 133 or 134
136. Audiology.mp.
137. exp Audiology/
138. audiologist.mp. or exp Audiologists/
139. deaf.mp.
140. Deaf.mp.
141. deafness.mp. or exp Deafness/
142. “deaf blind disorders”.mp. or exp Deaf-Blind Disorders/
143. “persons with hearing impairment”.mp. or exp Persons With Hearing Impairments/
144. “hearing loss”.mp. or exp Hearing Loss/
145. “hard of hearing”.mp.
146. “hearing disorders”.mp. or exp Hearing Disorders/
147. “hearing impairment”.mp.
148. exp Communication Disorders/or “communication disorders”.mp.
149. “communication delay”.mp.
150. “communication impairment”.mp.
151. “communication sciences”.mp.
152. “communication therapy”.mp.
153. “speech therapy”.mp.
154. exp Speech Therapy/
155. “Speech Language Pathology”.mp. or exp Speech-Language Pathology/
156. “speech language therapist”.mp.
157. “speech and language”.mp.
158. exp Speech Disorders/or “speech disorders”.mp.
159. “speech-therapist”.mp.
160. “speech therapist”.mp.
161. “speech pathology”.mp.
162. “speech pathologist”.mp.
163. “speech-language pathologist”.mp.
164. “speech-language therapy”.mp.
165. “speech-language therapist”.mp.
166. “language disorder”.mp. or exp Language Disorders/
167. “language delay”.mp.
168. “language disability”.mp.
169. “language impairment”.mp.
170. “language development disorders”.mp. or exp Language Development Disorders/
171. exp Articulation Disorders/or “articulation disorders”.mp.
172. “articulation delay”.mp.
173. “articulation impairment”.mp.
174. “Developmental language disability”.mp.
175. “SLP”.mp.
176. 136 or 137 or 138 or 139 or 140 or 141 or 142 or 143 or 144 or 145 or 146 or 147 or 148 or 149 or 150 or 151 or 152 or 153 or 154 or 155 or 156 or 157 or 158 or 159 or 160 or 161 or 162 or 163 or 164 or 165 or 166 or 167 or 168 or 169 or 170 or 171 or 172 or 173 or 174 or 175 or 176
177. *46* and *89* and *117* and *135* and *177*
