# Supplementary material for: Barriers and facilitators to paediatric caregivers’ participation in virtual speech, language, and hearing services: A scoping review
Source: Digit Health. 2023 Nov 29;9:20552076231216684. doi: 10.1177/20552076231216684 (PMC10687955; doi:10.1177/20552076231216684)
Supplement: sj-docx-2-dhj-10.1177_20552076231216684 - Supplemental material for Barriers and facilitators to paediatric caregivers’ participation in virtual speech, language, and hearing services: A scoping review [file sj-docx-2-dhj-10.1177_20552076231216684.docx]

**Supplement B.** Data charting tool.

| Factor | Extraction details |
| --- | --- |
| Area of communication sciences and disorders? | Audiology, Speech Language Pathology, or both. |
| Research topic? | Description, including study design. |
| Data collection tool? | Description (e.g. survey, interviews). |
| Participant group? | Number of total participants and number of caregivers. |
| Description of caregiver participant? | Describe caregiver (parent, mother, caregiver, etc.). |
| Role of caregiver? | Include details available. |
| Age of participants? | Age of child and age of caregivers. |
| Who is receiving the intervention? | Description (child or child and parent). |
| Health care providers included? | Description (Speech Language Pathologist, audiologist, etc.). |
| Type of intervention? | Description (counselling, coaching, etc.). |
| 1-on-1 or group based? | State if 1-on-1 or group based. |
| Type of interaction? | Description (video, audio, face-to-face). |
| Quality of interaction? | Description of factors that influenced communication. |
| Time-point in care process? | Description (follow-up, initial, both). |
| Caregiver location? | Description (home, clinic, etc.). |
| Contextual factors influencing intervention? | If provided, please describe. |
| Virtual model? | Describe model (synchronous, asynchronous, hybrid). |
| Provider location technologies? | Describe technologies at provider location. |
| Caregiver location technologies? | Describe technologies at caregiver location. |
| Caregiver education level? | Include details available. |
| Caregiver employment status? | Include details available. |
| Caregiver comfort with technology? | Include details available. |
| Caregiver access to technology? | Include details available. |
| General technology issues reported? | Include details available. |
| Was caregiver readiness discussed? | Yes/No. If yes, please describe. |
| Caregiver previous experience with virtual tech and virtual care | Include details available. |
| Caregiver’s knowledge about their child’s disorder? | Include details available. |
| Were caregiver personality factors found to relate to success with virtual care? | Yes/No. If yes, please describe. |
| Were caregiver technology aptitude or skills formally assessed? | Yes/No. If yes, please describe. |
| Was caregiver training provided around technology use? | Yes/No. If yes, please describe. |
| Reported benefits to virtual care? | Include details available. |
| Reported challenges to virtual care? | Include details available. |
| Other outcomes of interest? | Include details available. |
| Was an assessment tool used to evaluate candidacy/readiness/engagement in or with virtual care? | Yes/No. If yes, please describe. |
